# A survey of the transmission of infectious diseases/infections between wild and domestic ungulates in Europe

**DOI:** 10.1186/1297-9716-42-70

**Published:** 2011-06-02

**Authors:** Claire Martin, Paul-Pierre Pastoret, Bernard Brochier, Marie-France Humblet, Claude Saegerman

**Affiliations:** 1 Research Unit in Epidemiology and Risk Analysis Applied to Veterinary Sciences (UREAR), Department of Infectious and Parasitic diseases, Faculty of Veterinary Medicine, University of Liège, Boulevard de Colonster, 20, B42, B-4000 Liège, Belgium; 2 Publications Unit, World Organisation for Animal Health (OIE), 12 Rue Prony, 75017 Paris, France; 3 Scientific Institute of Public Health, Department of Microbiology, Division of Virology, Rue Juliette Wytsman 14, B-1050 Brussels, Belgium; 4 Anses, Sophia-Antipolis Laboratory, Unit of Ruminant Pathology, 105 Route des Chappes B.P.111, 06902 Sophia Antipolis Cedex, France

## Abstract

The domestic animals/wildlife interface is becoming a global issue of growing interest. However, despite studies on wildlife diseases being in expansion, the epidemiological role of wild animals in the transmission of infectious diseases remains unclear most of the time. Multiple diseases affecting livestock have already been identified in wildlife, especially in wild ungulates. The first objective of this paper was to establish a list of infections already reported in European wild ungulates. For each disease/infection, three additional materials develop examples already published, specifying the epidemiological role of the species as assigned by the authors. Furthermore, risk factors associated with interactions between wild and domestic animals and regarding emerging infectious diseases are summarized. Finally, the wildlife surveillance measures implemented in different European countries are presented. New research areas are proposed in order to provide efficient tools to prevent the transmission of diseases between wild ungulates and livestock.

## 1. Introduction

### 1.1. General introduction

The transmission of infectious diseases between wild and domestic animals is becoming an issue of major interest [1]. Scientists still lack of knowledge concerning the means and ways a large majority of infectious agents are transmitted. Wildlife can be exposed to domestic animal diseases resulting in severe consequences on their populations. On the other hand, numerous emerging infectious diseases (EIDs), including zoonoses, were shown to originate from wildlife [2,3]. Multiple publications dealing with wildlife diseases focus on zoonoses, while the present review targets the wild ungulates present in Europe (focussing on *suinae *and ruminants [4]), considering their close ecological and phylogenic relationship with livestock. The main objectives of this review are (i) for the first time, to establish a list as complete as possible of infectious agents already reported in European wild ungulates, (ii) to evaluate the possible role of both wild and domestic ungulates in the transmission of infectious diseases and (iii) to emphasize the importance of considering wildlife when studying the epidemiology of infectious diseases. Indeed, wild species may be infected by livestock pathogens and, at the same time, be a risk for the re-infection of livestock [5]. Thus, their importance in global animal health and in farming economy must be taken into account. This review is the first to list so exhaustively infectious diseases/infections already reported in European wild ungulates and, above all, to address their potential epidemiological role (e.g. reservoir, spillover, dead-end host and asymptomatic excretory animal). Bacterial, viral and prion, parasitic diseases are listed in three additional files (additional file 1, additional file 2 and additional file 3). In order to better understand the epidemiology of diseases/infections at the domestic animals/wildlife interface, global risk factors associated with the transmission of infectious diseases are reviewed. Finally, the different measures implemented by European countries regarding wildlife diseases/infections are summarized and new areas of research are suggested.

### 1.2. Methodology of bibliographic research

A list of bacterial, viral and parasitic diseases known to affect wild ungulates or livestock in Europe was established. The starting point was the list of diseases reportable to the World Organization for Animal Health (OIE). A bibliographical research was performed, combining the [name of pathogens] or the [name of the disease associated] with [ungulate] or [wildlife] or [wild ungulate] on web medical servers and databases (Medline, PubMed, CAB abstracts and ISI Web of Knowledge). Researches on prevalence or seroprevalence studies were mostly carried out from October 2008 to March 2009. No time limits of publication were imposed. For each pathogen, the most recent publications covering a maximum of European countries were selected. Furthermore, for each risk factor or perspective considered, a bibliographic review was launched in both Pubmed and ISI Web of Knowledge databases to identify the most suitable publications (fitting with keywords introduced, and illustrating problematic of concerns).

## 2. Current situation/status of European wild ungulates

### 2.1. Species and countries of concerns

This review targets wild ungulates present in the European continent (not only the European Union). They are listed in Table 1 according to their phylogenic relationship. Data about the origin of populations (natural vs. introduced) as well as their geographical distribution are adapted from a recently edited book [6].

**Table 1:** Classification, origin of the populations and geographical distribution of ungulates presents in Europe (from [5])

*Family*	*Sub-family *	*Species *	Latin name	Natural/introduction	European location
Suidae		Wild boar	*Sus scrofa*	Natural populations Introductions in Great Britain	All European countries

Cervidae	Cervinae	Chital	*Axis axis*	Introductions	Croatia, Istrian peninsula
		
		Fallow deer	*Dama dama*	Introductions Almost all populations are farmed animals.	All European countries
		
		Red der	*Cervus elaphus*	Natural populations Introductions in Corsica Introduction in Sardaigna	All European countries
		
		Sika deer	*Cervus **n**ippon*	Introductions in the XIX^e ^century	Northern Europe
		
		Reeves' muntjac	*Muntiacus reevesi*	Introductions in beginning of XXe century (native from China)	Great Britain
	
	Hydropotinae	Chinese water deer	*Hydropotes inermis*	Introductions	Great Britain
	
	Capreolinae	European roe deer	*Capreolus capreolus*	Natural populations	All European countries
		
		Elk	*Alces alces*	Natural populations	Northern Europe
		
		White-tailed deer	*Odocoileus virginianus*	Introductions (native from North America)	Finland, Czech Republic, Serbia, Croatia
		
		Reindeer	*Rangifer tarandus*	Natural populations Introduction in Iceland	Scandinavia Iceland

Bovidae	Bovinae	European bison	*Bison bonasus*	Natural populations or reintroductions	Central Europe (Poland, Byelorussia, Lithuania, Ukraine)
	
	Caprinae	Barbary sheep	*Amnotragus lervia*	Introductions	Spain
		
		Muskox	*Ovibos moschatus*	Introductions	Norway, Greenland
		
		Mouflon	*Ovis gmelinii*	Natural populations and introductions	All central and South of Europe
		
		Alpine chamois	*Rupicapra rupicapra*	Natural populations	Alpine mountains
		
		Pyrenean chamois	*Rupicapra pyrenaica*	Natural populations	Pyrenean mountains (France and Spain) Cantabric mountains (Spain) Abruzzi (Italia)
		
		Wild goat	*Capra aegragrus*	Introductions	Mediterranean islands (Balearic Islands, Crete)
		
		Alpine ibex	*Capra ibex*	Natural populations and reintroductions	Alpine mountains (France, Switzerland, Italy)
		
		Spanish ibex	*Capra pyrenaica*	Natural populations and reintroductions	Mountains of Spain and Portugal

### 2.2. Definition of important concepts

#### 2.2.1. Definition of an infectious disease/infection

The definition of an infectious disease/infection is the first step towards understanding the mechanisms involved in the transmission of a pathogen between animals. The first definition was given by Koch in four postulates at the end of the 19^th ^century. However, they are stated in a "one disease-one agent" model and are almost exclusively based on laboratory considerations. Several characteristics such as carrier state, opportunistic agents or predisposing factors are not taken into account with this definition. A disease may be currently defined as "any perturbation, not balanced, of one or more body function(s)" [7], which includes responses to infectious as well as non infectious agents [8]. In wild animals, characterized by feeding, reproduction and movements mostly independent from human activities (in opposition to domestic animals) [9], disease is strongly associated with environmental factors. Ecological factors are of major importance in the dynamics of wild populations as their survival rate and fecundity may be influenced by diseases [8]. A new concept of disease ecology recently emerged. For a well defined target population, the study of a disease/infection should be related to the study of interactions between the environment, pathogens and human activities [1,10]. For practical reasons, in this review, the term disease will be used to design both disease and infection.

#### 2.2.2. Definitions of epidemiological roles

Studying and controlling an infectious disease implies the knowledge of all actors involved in its transmission. A reservoir, or maintenance host, "is able to maintain an infection in a given area, in the absence of cross-contamination from other domestic or wild animals" [11]. Some authors distinguish different types of reservoirs (1) true reservoir (the species alone maintains the infection), (2) accessory reservoir (maintains the infection secondarily to the main reservoir), (3) opportunistic reservoir (accidentally infected, but without serious consequences) and (4) potential reservoir (can be a reservoir for biological or ecological reasons, but, to date, has not been identified as such under field conditions) [7]. For each category, the reservoir is related to a target population [12]. Spillover hosts can maintain the infection after recurrent contacts with an external source [11]. However, the categorisation of a species is not definite and may be a question of time: the integration in the maintenance or spillover categories of hosts is dynamic as a spillover species may become a reservoir as suspected in the French Brotonne forest: cervids were initially spillover hosts for *Mycobacterium bovis *but because of a high density of animals, the infection spread among them and they now act like maintenance hosts [13]. Wildlife pathogens can also spill back to domestic animals [3]. A dead-end host may be infected by a pathogen but does not allow its transmission in natural conditions; such status may be lost by a species under modified environmental conditions [7]. Finally, an infected animal can excrete a pathogen without showing obvious clinical signs. It is important to mention that the environmental survival of pathogens may also determine wether or not an asymptomatic excretory animal may be considered as reservoir.

Although definitions seem to be clearly delimited, it is not so easy to determine the particular role of a species. Indeed, out of 295 descriptions of wildlife infections reported in the additional files, their epidemiological role is only suggested by the authors in 34.2% of cases (N = 101). Authors often lack of data concerning species interactions as well as the infection status in other species. Besides, to determine the epidemiological role of a wild species towards domestic animals, it is required to assess the real status of livestock, which might not be always the case [14].

### 2.3. Review of some infectious diseases already reported in European wild ungulates

A global view of infectious diseases affecting domestic animals but already reported in European wild ungulates is presented in additional file 1 (bacteria), additional file 2 (viruses and prions) and additional file 3 (parasites). The epidemiological role of each species with respect to the pathological agent is specified. Nevertheless, it is not an exhaustive list of all diseases affecting wild ungulates as these studies only focused on pathogens affecting domestic animals. Pathogens were generally characterized by laboratory tests developed for domestic livestock. Some results such as apparent prevalence may therefore be biased [14]. In addition, the achievement of studies will also largely depend on the geographical accessibility of the region [15].

## 3. Risks factors associated with the transmission of diseases

**Figure 1:**
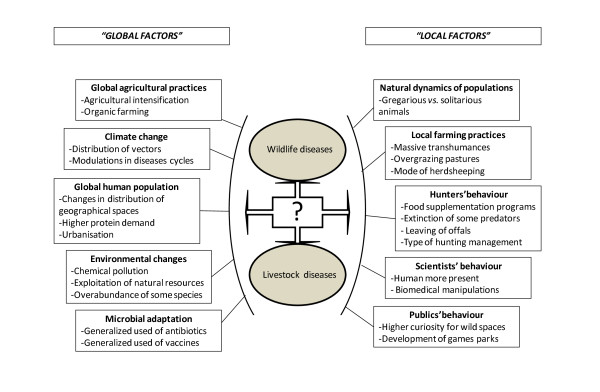
**Spatial classification (local vs. global) of the main factors involved in the transmission of pathogens between wild and domestic ungulates**.

A wide range of factors related to the ecology of diseases, e.g. environmental and ecological parameters, are constantly changing and will subsequently induce modifications in the transmission of pathogens. According to the OIE *Terrestrial Animal Health Code*, an EID is "a new infection resulting from the evolution or change of an existing pathogenic agent, a known infection spreading to a new geographic area or population, or a previously unrecognised pathogenic agent or disease diagnosed for the first time and which has a significant impact on animal or public health" [16]. Approximately 75% of the pathogens having affected or affecting humans for the last 20 years originate from animals [17]. Moreover, 72% of human EIDs reported between 1940 and 2004 find their origin in wildlife [18]. The role of wild ungulates as a reservoir of infectious diseases, for both humans and livestock, is now well established [19]. Over 250 species of human pathogens have been isolated from ungulates [20]. The main factors affecting the transmission of pathogens among populations of wild ungulates are listed hereafter. Factors related to the host, the pathogen and the environmental changes are considered separately [21]. Most environmental modifications are anthropogenic because directly or indirectly linked to human activities, thus, they are expected to change with time [3]. A spatial classification (local vs. global) of the main factors involved in the transmission of pathogens between wild and domestic ungulates are illustrated in Figure 1.

### 3.1. Global level (national or European level)

#### 3.1.1. Environmental changes

##### 3.1.1.1. Distribution of geographical spaces

Different factors can explain the constantly increasing interactions between wild and domestic animals. A major parameter is the growing human population, which increased four times during the previous century to now reach 6.9 billion people [22]. Such human population involves a huge and diversified protein demand constantly increasing [23]. In most European countries, large populations of wild ungulates are concentrated in small delimited areas because of high human distribution and densities. Degradation and fragmentation of wild spaces are the main anthropogenic factors associated with the emergence of diseases in wildlife [10,24]. The Food and Agricultural Organization (FAO) website [25] provides surface areas of the different type of land cover (agricultural, forestry, crops, meadows, etc.) since 1961 for almost all European countries: their evolution rates in Europe are summarized in Table 2. Until the nineties, areas dedicated to permanent crops and permanent pastures were increasing, leading to a diminution of natural landscape available for wild animals. However, a recent increase in forests areas as well as a global reduction of agricultural areas are observed, reflecting a decreasing importance of agriculture in the economy and additional space for wild populations (positive for wildlife conservation). What will be the real impact on the transmission on infectious diseases between wild animals is still to be assessed.

**Table 2:** Evolution of European lands resources

	1990/1961	2008/2000
Country area	1.000	1.000

Agricultural area	0.993	0.967
Arable land	0.935	0.964
Arable land and Permanent crops	0.939	0.963
Fallow land	*	*
Forest area	*	**1.005**
Inland water	1.003	1.008
Land area	1.000	1.000
Other land	*	1.014
Permanent crops	**1.024**	0.948
Permanent meadows and pastures	**1.047**	0.973
Temporary crops	*	*

Ratios (i) equal 1 mean that the area stayed constant during the period considered (ii) lower than 1: diminution of the area (iii) higher than 1: augmentation of the area concerned.

These ratios were obtained dividing land areas (in 1000 Ha) of 2 years. We performed 2 ratios, [area in 1990]/[area in 1961] and [area in 2008]/[area in 2000], to have a constant total European countries area (which changed between 1990 and 2000).

*unavailable data

Data obtained from the fao website, consulted 19 December 2010 (updated on September 2010). http://faostat.fao.org/site/377/DesktopDefault.aspx?PageID=377#ancor. Request was effectuated with the selection: (i) Country: "Europe + (Total)" and "Europe > (List); (ii) Year: "1961, 1970, 1980, 1990, 2000, 2008"; (iii) Item: "Country area, Agricultural area, Arable land, Arable land and Permanent crops, Fallow land, Forest area, Inland water, Land area, Other land, Permanent crops, Permanent meadows and pastures, Temporary crops".

##### 3.1.1.2. Chemical pollution

Chemical pollution may have a negative impact on wildlife demography or disease susceptibility. Direct impact on reproductive parameters and sex ration has been described [24]. Immunodepression can directly result from a toxic accumulation of chemicals at subclinical levels and increase the susceptibility to infectious diseases [26]. Several studies targeting the consequences of chemical pollution on wildlife reported a direct negative impact on birds and rodents but only few studies focused on wild ungulates [27]. In France, wildlife intoxication reports are registered by the SAGIR Network, in charge of the wildlife health surveillance [28]. Twenty five percent of mammalian intoxication reports concerned ungulates, but only 2.1% of cases were confirmed by positive findings [27]. Scientists reported a biomagnification of chemical concentrations via a food-chain transfer: for instance, liver concentrations of chlordecone, a carcinogenic insecticide, were lower in herbivores (bottom of the food chain) than in carnivores, and concentrations in scavengers were still more elevated (top of the food chain) [29]. The season of sampling should be considered whenever using wildlife as an accumulative bioindicator of environmental pollution. Indeed, seasonal variability in metal levels measured in roe deer kidneys found its origin in the difference of nutrition, both quantitative and qualitative. Seasonal peaks for the majority of metals are observed in a very narrow period (summer-autumn). Some plant taxons, such as fungi, are an important pathway for heavy metal intake into the mammalian organism [30]. In addition, consequences and interactions of chemicals on the expression of a disease are not entirely elucidated yet.

#### 3.1.2. Global agricultural practices

The last century was marked by an evolution of agricultural practices especially through industrialisation. Until the nineties, populations of "European classic livestock species" (cattle, sheep, goat, pig) were globally increasing (Table 3), along with an increase of areas dedicated to farming (Table 2). In such systems, domestic animals were genetically selected for a specific production, and as a result, they are less hardy and resistant to a high exposure rate of pathogens. However, since a few years, everywhere in Europe, public opinion is getting worried about the environment: people are in favour of an agriculture respectful of the environment. Development of organic farming is thus gaining much interest: areas dedicated to such farming were occupying more than 6% of the total agricultural areas in 2008 in Europe [25]. In opposite to the global intensification of agricultural practices, extensive farming systems regain interest, facilitating contacts between livestock and wildlife.

**Table 3:** Evolution of the number of living animals in Europe

	1970/1961	1980/1970	1990/1980	2000/1990	2009/2000	Global rate 2009/1961
**Cattle**	**1.13**	**1.15**	0.98	0.60	0.85	0.65
**Goats**	0.76	**1.01**	**1.28**	0.86	0.84	0.71
**Pigs**	**1.11**	**1.33**	**1.05**	0.77	0.94	**1.12**
**Sheep**	0.96	**1.04**	**1.11**	0.50	0.89	0.49
**Donkeys**	0.69	0.72	0.81	0.59	0.79	0.19
**Buffaloes**	0.89	0.85	**1.04**	0.40	**1.49**	0.47
**Camels**	0.86	0.97	**1.11**	0.04	0.70	0.02
**Horses**	0.70	0.72	0.92	0.69	0.90	0.29
**Mules**	0.57	0.52	0.63	0.70	0.85	0.11

Ratios (i) of 1 mean the numbers remained constant during the period of concern (ii) ratios < 1: decreased number (iii) and > 1: increased number. These ratios were obtained by dividing numbers of animals aged 2 years.

Data obtained from the fao website, consulted 19 December 2010 (updated on September 2010). http://faostat.fao.org/site/573/default.aspx#ancor. Request was effectuated with the selection: (i) Country: "Europe + (Total)" and "Europe > (List); (ii) Year: "1961, 1970, 1980, 1990, 2000, 2009"; (iii) Item: "Cattle, Goats, Pigs, Sheep, Asses, Buffaloes, Camels, Horses, Mules".

#### 3.1.3. Microbial evolution and adaptation

Pathogens lacking intermediate stages such as viruses, bacteria or protozoans are the main recently emerged pathogens of wildlife [15]. Out of 31 pathogens identified as having a real impact on the dynamics of mammals, 41% are viruses [31]. Because of their high mutational rate, RNA viruses are perfect candidates for emergence. However, even if the evolution of pathogens plays a key role in the emergence of diseases, the ecological factors described below also favour their emergence [26].

#### 3.1.4. Climate change

According to the last report of the Intergovernmental Panel on Climate Change (IPCC), the earth's surface and oceans temperatures are increasing by leading to the constant reduction of land snow cover and the melting of sea ice and glaciers [32]. The global mean surface air temperature increased of an average of 0.75 C since the mid-twentieth century and climate experts expect this increase to continue during the 21th century [33]. As a result, changes in ecosystems are occurring in many parts of the world: the distribution of species and timing of events in some seasonal cycles are affected [34]. In Europe, changes are less obvious than in other sensible parts of the world such as arctic or tropical ecosystems. However, epidemiological cycles are affected since the temperature threshold may modulate the cycle of vector-borne microorganisms [35]. Climate changes might favour the emergence of vector-borne diseases and be responsible of outbreaks of known diseases in regions where they were never reported before. The prevalence and distribution of well-known vector-borne diseases have already increased during the last decade [33]. In the Mediterranean region, bluetongue virus (BTV) recently emerged and became enzootic in livestock [36]. Wild ungulates were proved to be receptive to the virus in all European regions [37,38]. In southern Spain, BTV antibodies were detected in wild ruminants in areas where no outbreak had been reported in livestock, suggesting their potential role of reservoir for BTV, but this statement requires further confirmation [39]. The distribution of ticks is evolving along with climate changes. Indeed, during the last 20 years, the upper limit of tick distributions shifted from 700-800 m to 1200-1300 m above the sea level [40]. Consequences on wildlife infections were immediate: in 2005, tick-borne babesiosis was reported for the first time in chamois (*Rupicapra rupicapra*) in Switzerland [41].

#### 3.1.5. Global increased mobility and trade

The last decades were marked by an increased human and animal mobility as well as a constantly evolving animal trade. The translocation of wild or domestic animals is one of the major factors responsible for the introduction of diseases. The trade of living animals was multiplied by a factor 10 between 1995 and 2005: global imports and exports were respectively 8.8 and 13.5 times more important in 2005 than in 1995 [42]. Transports are often carried out under very poor conditions because animals are piled up and stressed. Their susceptibility to infections increases. Even if it mainly concerns species other than ungulates, wildlife trade is one of the main problems in a potential cross-species transmission of infectious agents [43]. One should also consider (re)introduction of wild animals for hunting purpose when focusing on wildlife trade. The presence in Europe of most non-native species of ungulates may be explained by such practices. It is currently almost impossible to quantify the global wildlife trade as it is mostly illegal. However, the economic impact resulting from outbreaks caused by wildlife trade has globally reached hundreds of billions dollars to date [23]. Spatial mobility of humans was multiplied by more than 1000 since 1800. A 222% increase is expected for the number of passenger per km by 2035 [44]. As the incubation period of most infections exceeds the time necessary to transfer an animal from a country to another [45], the propagation of pathogens and vectors has reached an unprecedented rate.

### 3.2. Local level (regional or district)

#### 3.2.1. Natural dynamics of populations

The social organisation of populations impacts the transmission rate of infections: the probability of contacts is higher for gregarious animals than for solitary species. Besides, the reproduction period is characterised by increased contacts between individuals [9]. Furthermore, the exposure to pathogens depends on the presence/absence of migratory flows [3]. European wild ungulates are not migratory animals as such, except reindeer (*Rangifer tarandus*). Nevertheless, once wild populations colonize and occupy a given area, some animals might later radially disperse to close areas and be at risk for contamination [5]. Natural and artificial barriers are likely to limit animal movements and may thus reduce the transmission of pathogens.

#### 3.2.2. Human behaviours

Contacts between wildlife and livestock are also increasing because behaviours of farmers, hunters, scientists and the general public are changing.

##### 3.2.2.1. Farmers

Along with a global change of agricultural practices at the European scale, it is important to consider local agricultural practices. Changes of farmers' behaviours mostly impact contact rates between wild and domestic ungulates. Pastures are places where the transmission rate of infectious diseases is the highest [46]. Farmers' management of pastures are thus of major importance. Some practices such as salt deposits in alpine pastures enhance the risk of indirect transmission of pathogens, like *Pasteurella *for example [47]. Mountain transhumance (summer moving of domestic flocks to alpine meadows) was initially performed at walking-distance. Nowadays, flocks are moved by cattle-trucks, allowing long-distance transportations of more animals; alpine meadows are overgrazed and the probability of contacts with wildlife increases. Besides, whereas initially created to protect biodiversity, national parks allow domestic flocks to graze inside their central part in some countries, which may have detrimental effects for both sides.

##### 3.2.2.2. Hunters

Hunting behaviours may play a major role in the transmission of diseases between or among wild populations. Food supplementation programs implemented to increase the number of hunting bags have drastically disturbed the natural regulation and spatial distribution of populations. Various wild populations, e.g. wild boar [48] or red deer [49], are constantly growing. For example, in Wallonia (Belgium), red deer and roe deer populations have increased twofold while wild boar populations have more than tripled between 1980 and 2005 [50]. In some other European areas, populations are overabundant. The hunting of predators led to their extinction and a subsequent imbalance of interactions between species. Offals of dead wild ungulates are generally left in the field, which may reach at the European scale thousands of tons of potentially infected materials in free access to other species. When an infectious disease is prevalent in wild populations, directed shots of sick animals are often applied. However, during a recent outbreak of infectious keratoconjonctivitis in Alpine wild ungulates, such measure seems to have prevented the natural immunisation of populations (Gauthier, personnal communication). A global reduction in hunting pressure may therefore be preferred, especially to protect reproductive adults.

##### 3.2.2.3. General public

For many city dwellers, contacts with nature are limited to controlled areas such as national parks or wildlife game parks. National/regional natural areas are government parks, of which the first objective is to protect natural lands (ecosystems). Wild ungulates may or may not be hunted in function of local legislation. In these opened parks, public frequentation is constantly increasing, as people are in search of a closer contact with nature under protected conditions. The frequency of contacts between wild species and humans increases as a consequence of natural tourism [51]. Wildlife game parks could be associated to 'game zoos': species belonging to the native European wild fauna are parked in closed areas. Densities of populations are often high and animals are frequently translocated between different parks. The high density rate can be implicated in the transmission of diseases [52]. Deer farming is promoted by several European governments like Switzerland [53]. In France, 400 deer farms are inventoried [54]. The proximity of several species (including humans) will subsequently play a key role in the contact rate.

##### 3.2.2.4. Scientists

More and more scientific studies focus on monitoring of wild populations. Even if carefully controlled, the intrusions of scientists may be a risk of disease transmission. Even if some introduction programs prevent animal transfers from one region to another, or between different countries, some wounded animals are brought to health cares and released after successful treatment. While it mainly concerns wild species other than ungulates, such practices can also increase the risk of diseases transmission.

## 4. Control measures of infectious diseases already implemented in European wildlife

The section below develops the measures already implemented or to be implemented by European countries to control the transmission of diseases between wild and domestic animals, at three different levels: (i) European; (ii) national, (iii) regional (local).

### 4.1. At European level

The continuity between all living beings involved in the transmission of infectious diseases must be treated from an international point of view.

#### 4.1.1. Wildlife-livestock-human continuum

As previously described, the importance of contacts between wildlife, livestock and humans is such that some authors suggested a "wildlife-livestock-human continuum" [55]. In 2008, King suggested to use the term "interdependence" instead of "independence" of these three compartments [56]. As a consequence, a new concept of conservation medicine emerged for the protection of animal, human and ecosystem healths [57]. The main goals are to promote the development of scientific studies for problems occurring at the interface between environmental and health (human and animal) sciences [58]. In this context, studies of the community ecology should be performed, in order to better understand the epidemiological links between all actors of the wildlife-livestock-human-continuum [59].

#### 4.1.2 Biodiversity and wild heritage

As already mentioned, infectious diseases affecting wildlife have several impacts such as depletion of populations and rare species (on their own or in concert with other factors) but management actions also have an environmental impact [60]. Nevertheless, if diseases are a risk for wildlife conservation, preserving biodiversity helps also avoiding their emergence. For example, the prevalence of vector-borne diseases will decrease if the variety of food sources (native hosts) increases, as the infestation rate within each species will be reduced [61].

##### 4.1.2.1. Wild mammals

The first modern complete inventory of mammals was established in 1982, with a list of 4 170 species identified (cited in [62]). The 1993-inventory included 4 629 different species [63]. In 2005, the complete list of mammals indexed 5 416 species the total number being estimated at around 5 500: 99% of mammalian species are thus probably already known [64]. Such increasing number of identified species is due to the separate listing of newly discovered phenotypes and genotyping through molecular biology (taxonomic revision). Two hundred and forty species of *Artiodactyla *pertaining to 89 genera are described, most of them living in the biodiversity "hot spots" located in Sub-Saharan Africa. European species of *Artiodactyla *are by contrast less numerous (see Table 1).

##### 4.1.2.2. Domestic species

Through selection, man created numerous breeds of domestic animals, e.g. there are approximately 700 breeds of cattle identified worldwide [65]. Nevertheless, many of them are on the verge of extinction, decreasing the genetic variability of cattle.

##### 4.1.2.3. Role of biodiversity in disease ecology

The influence of human activities on endangered and unmanaged wild fauna is of major concern. Out of 31 cases of disease emergence in wildlife, only 6 were not influenced by humans [15]. Eighty-eight percent of mammals at risk for severe infections and listed by the International Union for Conservation of Nature (IUCN) Red List of Threatened and Endangered Species are carnivores or artiodactyls [31]. Most livestock and companion animals belong to these categories. The degradation of ecosystems, the loss of habitats and diminishing food resources force some species to use alternative alimentary sources [1]. Biodiversity acts as a primordial barrier against infectious pathogens. Besides, anthropogenic factors causing losses of biodiversity increase the risk of disease emergence [26] by modifying the abundance, the behaviour or the condition of hosts or vectors [66]. It is then crucial to preserve biodiversity in an integrated and sustainable manner [67].

#### 4.1.3. OIE working group on wildlife diseases

In order to develop specific surveillance guidelines for wildlife diseases, the OIE recently created a Working Group on Wildlife Diseases [68]. It provides information on the wild animal health status, either in the wild or in captivity. Its most important missions are: (i) the elaboration of recommendations and the reviewing process of scientific publications on wildlife diseases; (ii) the implementation of surveillance systems of the wildlife-domestic animals-human continuum and (iii) the control of emerging and re-emerging zoonoses.

#### 4.1.4. Prioritization of wildlife diseases

Based on an OIE imported framework, a method of "rapid risk analysis" was developed in New Zealand with the aim to prioritize pathogens for the wildlife disease surveillance strategy [69]. Authors first listed all wildlife pathogens likely to interfere with animal or human health. They selected the pathogens likely to have a serious impact on wildlife, livestock and/or humans, after consulting experts of each sector. The risk estimate for each pathogen was scored on a semi-quantitative scale (from 1 to 4). The likelihood and consequences of spread were assessed for free-living and captive wildlife, livestock (distinction between consequences on productivity, welfare and trade), humans and companion animals. The risk of introduction in New Zealand was also assessed (scores: 0 or 1). Finally, pathogens were ranked and authors listed the top exotic and endemic dangerous wildlife pathogens for each population of interest (wildlife, domestic animals or human). Summing the risk estimate for each population gave a "total risk estimate" [69]. In Europe, the French agency for food, environmental and occupational health safety (Anses) multidisciplinary working group also elaborated a two-phase risk prioritization method [35]: (i) identification of diseases of which the incidence or geographical distribution could be affected by climate change, (ii) the risk assessment for each disease. Twenty diseases likely to be influenced by climate changes were selected. The authors qualitatively assessed the risk of each disease for its impact on human and animal health and on economy, considering the likelihood of disease evolution and the impact level. Three diseases affecting ungulates were selected for which some measures needed to be implemented (BTV, Rift Valley Fever and African horse sickness).

The prioritisation of diseases is useful to (re)-direct and target funds allocated to diseases surveillance and research. Organisms involved in wildlife conservation will be more inclined to financially support the control of wildlife diseases [69]. However, several current EIDs should in fact be considered as re-emerging [70]. To focus wildlife surveillance on prioritized agents could lead to a reduced vigilance/surveillance of "old" diseases. Their implementation in a global surveillance of wildlife diseases should be conducted carefully.

### 4.2. At country level

Some decisions will depend on the organization of national governments and bodies in charge of sanitary surveillance.

#### 4.2.1. Surveillance programs

Disease surveillance is defined by the World Health Organization (WHO) as "the ongoing systematic collection, analysis and interpretation of data but also the dissemination of information to the different actors involved in wildlife management" [71]. For the OIE, surveillance is "aimed at demonstrating the absence of disease/infection, determining the occurrence or distribution of disease/infection, while also detecting as early as possible exotic or emerging diseases" [72]. Several European Member States (MSs) have already implemented a health monitoring of their main wild populations. Surveillance systems of wildlife diseases are usually declined in passive surveillance, which consists in reports and necropsies of all animals found dead, and active surveillance, declined as the sampling of some populations in order to assess the (sero)-prevalence of infections. Such systems are now well developed in Belgium [37], Spain (Gortazar, personal communication), France (SAGIR Network) [73] and Switzerland (Ryser-Degiorgis, personal communication). A National Health Surveillance Program for cervids (HOP) was implemented in Norway in 2001 [74]. In Sweden, a monitoring of wildlife health exists since 1945 and became an integrated part of the National Environmental Monitoring Programs [75].

Such systems should be developed at a larger scale. Each State should be able to provide relevant information on the health status of its wild populations. To help other countries developing surveillance systems, it may be interesting to provide guidelines with different modalities in function of the specific epidemiological situation. Standardization of protocols between the different countries would permit a better global and harmonized evaluation of diseases status, and would allow the implementation of an efficient surveillance system. Moreover, the implementation of epidemiological surveillance should be based on both epidemiological (regular collection and analysis of epidemiological information and early warning systems for animal diseases) and ecological monitoring (surveillance of vectors and wild reservoirs) [35].

#### 4.2.2. Vaccination programs

Several reasons may justify the implementation of vaccination programs in wild animals: (i) conservation of endangered species, (ii) reduction of disease impacts, (iii) protection of human health (zoonotic agents) and (iv) prevention of transmission to domestic animals (and subsequent economic losses) [58]. Besides, vaccination is an alternative to global culling of wild reservoirs. However, it is important to keep in mind the goals of a vaccination programme. Indeed, a safe and effective vaccine can be used in restricted threatened populations and provide expected results. To eliminate a pathogen in a large area or in large populations, vaccination programs may be used in a multiple-hosts system or at a too-large scale and be unsuccessful. The majority of available vaccines have been developed for domestic animals, and their efficacy and safety are in most cases unknown for wildlife. An ideal vaccine for wildlife should be (i) administered *per os*, (ii) mono-dose (iii) safe for target and non-target species and, if possible, (iv) inexpensive to produce [76]. For example, in Europe, vaccination programs have been implemented in wild boar for classical swine fever (CSF). In France, a quantitative and retrospective study showed that a preventive vaccination (using oral baits) in a determined region improved the control of CSF, but did not eradicate the disease [77]. For multi-hosts pathogens such as *Mycobacterium bovis*, vaccination programs may be more difficult to implement [78], the previous identification of reservoir(s) being essential. Vaccination programs against *M. bovis *were recently started in the UK for badgers [76] or in Spain for wild boar [79]. In conclusion, vaccination programs can be used in wildlife under specific conditions, especially for small populations or in restricted areas [58].

#### 4.2.3. Sentinel animals

A sentinel species is an animal/species different from the target animal/species. The use of sentinel animals may be applied in three main situations: when adequate sampling of the target species is difficult (e.g. rare or endangered species), when the sentinel species is more abundant (e.g. use of sentinel chickens instead of wild birds for West Nile virus monitoring) and finally, when the species provides useful information on lower trophic level (e.g. the study of scavengers or carnivores) [8,80]. The place a species occupies in the food chain determines its probability of contamination [81]. The target and the sentinel population must be epidemiologically linked, at least spatially and the response of sentinel animals against a particular pathogen must be demonstrable [82]. For example, red deer are used as a sentinel species for the surveillance of BTV in Spain [38].

### 4.3. At local level (district or region)

(Inter)-national regulations must be implemented at local levels also, involving the participation of local structures, such as farmers groups or hunter organisations.

#### 4.3.1. Adaptation of livestock farming

Wild animals are often considered as reservoir of infectious diseases [19]. However, in many cases, infections originate from domestic animals. For instance, bovine herpesvirus 1 (BoHV-1) can induce a moderate infection in deer, whereas cattle is not at risk for the cervid herpesvirus 1 [83]. Thus, contacts should be limited but, at best, avoided between wild fauna and livestock [67]. In some regions of North America, brucellosis became endemic among wapitis (*Cervus elaphus*) and bisons (*Bison bison*). Bisons were infected by cattle around 1900, and the disease became endemic in those wild populations after their release. Although this example concerns non-European wild populations, the measures implemented are interesting to develop in this review. Despite the implementation of feedgrounds and vaccination, habitat improvement and prevention of commingling, livestock still remains infected. Other management options were then proposed: (i) removing cattle from public lands, (ii) developing and implementing brucellosis vaccines more effective for elks and bisons, (iii) managing cattle through vaccination and physical separation from elks and bisons and (iv) using contraceptives in elks to reduce pregnancies and abortions [84]. In the U.S. Sierra Nevada, a model assessing the impact of different management strategies of domestic sheep (grazing allotment closure, grazing time reductions and reduced probability of contact with stray domestic animals) on the transmission of respiratory diseases from domestic herds to endangered bighorn sheep was built [85]. In order to reduce the risk of disease transmission, the best solution was to avoid an overlapping between domestic sheep and bighorn sheep grazing areas.

Such epidemiologic studies show the importance of identifying and assessing the risks in order to implement preventive measures. Efforts should be devoted towards avoiding contacts between wild and domestic animals. Compartmentalisation and zoning are biosecurity measures advised by the OIE *Terrestrial animal health code *to avoid contacts between domestic and wild animals. However, such measures are often impossible to achieve in field conditions. The total surface area of the European continent occupied by national parks, protected zones where grazing is forbidden, is in fact very limited [83]. Efforts should be devoted to improve biosecurity in farms. In the UK, cattle often contract *Mycobacterium bovis *tuberculosis in pasture contaminated by badger excreta [86]. In order to reduce the risk of contamination in pasture, different practices such as the presence of ungrazed wildlife strips, and the greater availability, width and continuity of hedgerow may be proposed. The management of grazing has shown to reduce the risk of contamination. Here are other examples of efficient measures: rotational grazing system, off-fencing of setts and latrines, the avoidance of grazing pasture too short, the non-introduction of cattle to recently cut fields, the moving of cattle to fresh pasture in the afternoon and the absence of supplementary feeding on pasture [87].

#### 4.3.2. Specific hunting measures

While hunters may play an important role in the transmission of diseases, they can also be important for their control. Indeed, most scientific studies dealing with infectious pathogens in wildlife require an effective collaboration with hunters, as sampling is facilitated on carcasses of hunted animals. Such collaborations should be promoted at a larger scale. Besides, the establishment of controlled management plans for different known diseases should be promoted.

## 5. Perspectives

Interdisciplinary collaboration is a requisite to the success of management programs. Studies involving biologists, ecologists, veterinarians, epidemiologists and medical doctors should then be promoted. Nevertheless, further research is needed to clearly assess all consequences of the diseases transmitted between wildlife, livestock and humans. A better knowledge of wild populations (size and distribution) of each species should be promoted by applying harmonized methods among the different regions and/or countries. Besides, more studies could be performed in order to understand and analyse the infectious strains circulating among wild animals, but, above all, to compare them to strains circulating among domestic livestock. In most cases, researchers ignore if strains circulating among domestic and wild populations are similar. The epidemiological cycles of infectious diseases in all populations of concern are not well assessed to date. Then, it would be interesting to study methods of space sharing between wild and domestic animals. Costs associated as well as benefits for biodiversity and economical incentives for livestock farming should be evaluated. Because of numerous factors such as globalisation or climate changes, the threat of EIDs is clearly present. The impact of EIDs on economy and public health is not always easily predictable, and should receive more attention, through prioritization procedures for example. Awareness campaigns of politics via a direct estimation of costs generated by EIDs would allow funding research projects for wildlife health surveillance. Ecology and protection of the environment should also be integrated in research programmes without neglecting the surveillance of already known 'old' diseases.

To focus wildlife surveillance on prioritized agents could lead to a reduced vigilance/surveillance of "old" diseases. Their implementation in a global surveillance of wildlife diseases should be conducted carefully. The implementation of surveillance programs and research studies is not achievable without the involvement of local partners. However, the latter often complain about significant discordances between research (most of the time carried out at the European Union level) and field conditions (regional level). Awareness campaigns and a better communication between all sectors would ensure a better involvement of all surveillance actors and thus benefit to the global system. For example, the attribution of definite roles at the different levels would provide a more efficient distribution of work. Furthermore, information provided by the surveillance of wildlife should be available for the whole scientific community, in order to facilitate the development of spatio-temporal epidemiological methodologies to improve and refine it. Such approach would encourage interdisciplinary collaborations by involving all partners. Surveillance programs have already been implemented in wildlife such as the PREDICT project [88] developed by the Davis University of California: it uses a risk-based approach focused in areas where zoonotic diseases are most likely to emerge and where host species are likely to have significant interaction with domestic animals and high density human populations [88]. This proactive novel approach should be adapted to the specific EU situation. For some domestic species, epidemiologic networks are already in place, such as the RESPE network (Epidemiosurveillance Network of Equine diseases) in France [89]. This network is based on the existence of different specialized networks. It involves owners/farmers, veterinarians and laboratories. The role of each member is well definite, which comes out onto a well-working network. Besides, decisional trees may be suggested to local partners in order to adapt their management of wild populations and surveillance of diseases. These trees may propose different approaches for the populations' management in function of diseases or clinical signs reported. Such trees may simplify the decision making for local partners, when, for example, an epizooty starts in wildlife populations. Management plans will then be adapted more easily and more quickly.

A preliminary stage would be to categorise the diseases according to different parameters such as its mode of transmission, its pathogeny or the type of clinical signs it generates. Demographic specificities of the populations of interest (gregarious vs. solitary) must be taken into account also. According to the category of disease and the type of populations, management plans may be well adapted or not.

## 6. Conclusion

In 2004, King [45] reminded that knowledge and strategy were still missing for the prevention and control of wild animal diseases. Nowadays, governments and scientists become aware of the necessity to provide means for research on wildlife; scientific studies focusing on wildlife ecology as well as surveillance programs are indeed in expansion [1]. Nevertheless, numerous factors influencing the transmission and ecology of diseases reached a threshold without precedent, and are of major concern for the control of wildlife diseases, such as increasing pressure of humans on natural ecosystems and rising interactions between the different species. A better surveillance of wildlife diseases implemented in an integrated system involving international, national and local actors would be of major relevance to understand the origin of diseases and subsequently to control them. Efforts are required to reduce disagreements and misunderstandings between all actors involved in sanitary surveillance of wildlife. The preservation of biodiversity is crucial for diminishing the risk of disease transmission, as well as the improvement of farm biosafety.

## 7. Competing interests

The authors declare that they have no competing interests.

## 8. Authors' contributions

CM and CS participated in the conception and the design of the survey. CM carried out the majority of the bibliographic search and the redaction of the manuscript. PPP and BB revised the manuscript. MFH participated in the English improvement and in the revision of the manuscript. CS participated in the coordination as well as in the revision and commenting of the manuscript. All authors read and approved the final manuscript.

## Supplementary Material

Additional file 1 [90-137]. **Selected bacterial diseases reported in wild ungulates in Europe**. This file is a table presenting a list of bacterial diseases already reported in wild ungulates in Europe. Click here for file

Additional file 2 [138-171]. **Selected viral diseases reported in wild ungulates in Europe**. This file is a table presenting a list of viral diseases already reported in wild ungulates in Europe. Click here for file

Additional file 3 [172-202]. **Selected parasitic diseases reported in wild ungulates in Europe**. This file is a table presenting a list of parasitic diseases already reported in wild ungulates in Europe. Click here for file
