# Supplementary material for: A survey of the transmission of infectious diseases/infections between wild and domestic ungulates in Europe
Source: Vet Res. 2011 Jun 2;42(1):70. doi: 10.1186/1297-9716-42-70 (PMC3152899; doi:10.1186/1297-9716-42-70)
Supplement: Additional file 1 — [90-137]. Selected bacterial diseases reported in wild ungulates in Europe. This file is a table presenting a list of bacterial diseases already reported in wild ungulates in Europe. [file 1297-9716-42-70-S1.doc]

Additional file 1. Selected bacterial diseases reported in wild ungulates in Europe

| ***Pathogen*** | ***Ungulate specie***  ***(Latin name)*** | **n** | **N** | **Prevalence** | **Sero-prevalence** | **Diagnostic method** | **Epidemiological role from author’s opinion** | **Years** | **Country** | **Reference** |
| --- | --- | --- | --- | --- | --- | --- | --- | --- | --- | --- |
| *Anaplasma spp.*  (Anaplamosis) | *Capreolus capreolus* | 14 | 17 |  | X | ELISA | Reservoir | 2005 | Spain | [90,90] |
| *Anaplasma ovis*  (Anaplasmosis) | *Capreolus capreolus* | 9 | 17 | X |  | PCR | Reservoir | 2005 | Spain | [90] |
| *Anaplasma phagocytophilum*  (Anaplasmosis) | *Capreolus capreolus* | 23 | 72 | X |  | ELISA | Reservoir | 2003 | Poland | [91] |
|  |  | 18 | 132 | X |  | PCR | Unspecified | 2004 | Italy | [92] |
|  |  | ? | 56 |  | 94% | IFA | Unspecified | 1998 | Slovenia | [93] |
|  |  | 19 | 96 | X |  | PCR | Reservoir | 2001 | Italy | [94] |
|  |  | 52 | 121 | X |  | PCR | Reservoir | Unspecified | Austria | [95] |
|  |  | 217 | 227 |  | X | IFA | Reservoir | 2002-2003 | Denmark | [96] |
|  |  | 101 | 237 | X |  | PCR | Reservoir | 2002-2003 | Denmark | [96] |
|  |  | 1 | - | (case report) |  | PCR | Unspecified | 2004 | Norway | [97] |
|  | *Cervus elaphus* |  | 32 |  | 35% | IFA | Unspecified | 1998 | Slovenia | [93] |
|  |  | 15 | 150 |  | X | ELISA | Reservoir | 2000-2003 | Spain | [98] |
|  |  | 2 | 7 | X |  | PCR | Unspecified | Unspecified | Austria | [95] |
|  | *Bison bonasus* | 5 | 8 | X |  | Nested PCR | Reservoir | 2003 | Poland | [99] |
|  | *Dama dama* | 31 | 70 |  | X | IFA | Unspecified | 2004-2005 | Italy | [100] |
|  |  | 21 | 29 | X(µ) |  | PCR | Unspecified | 2004-2005 | Italy | [100] |
| *Borrelia burgdorferi*  (Lyme disease) | *Capreolus capreolus* | 83 | 227 |  | X | IFA | Unspecified | 2002-2003 | Denmark | [96] |
|  | *Rangifer tanrandus(&)* | 1 | 13 |  | X | ELISA | Unspecified | 1969-1998 | Germany | [101] |
|  | *Alces alces (&)* | 2 | 13 |  | X | ELISA | Unspecified | 1969-1998 | Germany | [101] |
|  | *Ovis amnon (&)* | 3 | 18 |  | X | ELISA | Unspecified | 1969-1998 | Germany | [101] |
| *Brucella suis*  (Brucellosis) | *Sus scrofa* | 168 | 763 |  | X | ELISA | Zoonotic reservoir | 1995-1996 | Germany | [102] |
|  |  | 98 | 424 |  | X | RBT+CFT  ELISA | Possible reservoir | 2003-2004 | Croatia | [103] |
| *Brucella suis biovar 2*  (Brucellosis) | *Sus scrofa* | 6 | 93 | X |  | Isolation | Possible reservoir | 2003-2004 | Croatia | [103] |
|  | *Sus scrofa* | 198 | 1841 | X |  | Bacteriology test | Unspecified | 2001-2007 | Italy | [104] |
| *Brucella suis biovar 3* | *Sus scrofa* | 1 | 93 | X |  | Isolation | Possible reservoir | 2003-2004 | Croatia | [103] |
| *Brucella melitensis biotype 2* | *Capra ibex* | 1 | 7 | (case report) |  | Isolation | High seroprevalence (4%) present among domestic animal in the area | 1996 | Italy | [105] |
| *Brucella melitensis biotype 3* | *Rupicapra rupicapra* | 1 |  | (case report) |  | Isolation | Sporadic case | 1988 | France | [106] |
| *Brucella spp.*  (Brucellosis) | *Cervus elaphus* | 2 | 54 |  | X |  | Unspecified | Unspecified | France | [107] |
|  |  |  | 5821 |  | 0.4 [0.3-0.6] | ELISA | Not reservoir | 1999-2009 | Spain | [108] |
|  | *Rupicapra pyrenaica* |  | 1410 |  | 0.8 [0.4-1.4] | ELISA | Not reservoir | 1999-2009 | Spain | [108] |
|  | *Capra pyrenaica* |  | 1086 |  | 0.1 [0-0.6] | ELISA | Not reservoir | 1999-2009 | Spain | [108] |
|  | *Capreolus capreolus* | 0 | 696 |  | X | Unspecified | Unspecified | Unspecified | France | [107] |
|  | *Sus scrofa* | 180 | 1821 |  | X | ELISA | Unspecified | 2001-2003 | Switzerland | [109] |
|  |  | 15 | 342 |  | X | ELISA | Unspecified | 2005-2006 | Italy | [110] |
|  |  |  | 4454 |  | 33 [31.6-34.4] | ELISA | Possible threat | 1999-2009 | Spain | [108] |
|  |  | 448 | 2267 |  | X | RBT + CFT | Unspecified | 2001-2007 | Italy | [104] |
|  |  | 62 | 211 |  | X | RBT + CFT | Unspecified | 1996-2000 | Croatia | [103] |
| *Campylobacter jejuni* | *Capreolus capreolus* | 1 | 38 | X |  | Culture | Unspecified | 2002 | Norway | [74] |
| *Chlamydia spp.* | *Capreolus capreolus* | 5 | 155 |  | X | Complement fixation | Unspecified | 1979 | France | [111] |
|  | *Bison bonasus* | 28 | 60 |  | X | CFT | Unspecified | 1980-1983 | Poland | [112] |
| *Chlamydia abortus* | *Sus scrofa* | 2 | 14 | X |  | PCR + sequencing | Possible reservoir | 2002 | Germany | [113] |
| *Chlamydia psittaci* | *Sus scrofa* | 4 | 14 | X |  | PCR + sequencing | Possible reservoir | 2002 | Germany | [113] |
| *Chlamydia suis* | *Sus scrofa* | 2 | 14 | X |  | PCR + sequencing | Possible reservoir | 2002 | Germany | [113] |
| *Chlamydophila pecorum* | *Rupicapra rupicapra* | 1 | - | (case report) |  | Isolation | Unknown | Unspecified | Italy | [114] |
| *Chlamydophila abortus* | *Capra ibex* | 3 | 306 |  | X | ELISA | Unspecified | 2006-2008 | Switzerland | [115] |
| *Coxiella burnetti*  (Q fever) | *Capreolus capreolus* | 3 | 175 |  | X | Complement fixation | Unspecified | 1979 | France | [111] |
|  |  | 4 | 78 | X |  | PCR | Unspecified | 2001-2006 | Spain | [116] |
|  |  | 6 | 39 |  | X | IFA | Possible reservoir | 2004-2005 | Spain | [117] |
|  | *Cervus elaphus* | 1 | 54 |  | X | Unknown | Unspecified | 1982-1985 | France | [118] |
|  |  | 34 | 116 |  | X | IFA | Possible reservoir | 2004-2005 | Spain | [117] |
|  | *Bison bonasus* | 7 | 60 |  | X | CFT + MAT | Unspecified | 1980-1983 | Poland | [112] |
|  |  | 36 | 47 |  | X | Unknown | Endemic disease | Unspecified | Poland | [119] |
|  | *Capra ibex* | 8 | 269 |  | X | ELISA | Unspecified | 2006-2008 | Switzerland | [115] |
|  | *Sus scrofa* | 4 | 93 | X |  | PCR | Unspecified | 2001-2006 | Spain | [116] |
| *Escherichia coli* | *Cervus elaphus* | 3 | 206 | X |  | PCR | Reservoir | 2005-2006 | Spain | [120] |
| *Francisella tularensis*  (Yersiniosis) | *Sus scrofa* | 24 | 763 |  | X | ELISA | Zoonotic reservoir | 1995-1996 | Germany | [102] |
| *Foot necrobacillosis complex (#)* | *Rangifer tarandus tarandus* | 100 | 3000 | X |  | bacteriological examination + PCR | Independent cases | 2007 | Norway | [121] |
| *Leptospira interrogans*  (Leptospirosis) | *Capra ibex* | 2 | 153 |  | X | MAT | Unspecified | 2006-2008 | Switzerland | [115] |
|  | *Sus scrofa* | 9 | 342 |  | X | MAT | Unspecified | 2005-2006 | Italy | [110] |
|  | *Bison bonasus* | 35 | 60 |  | X | MAT | Cross reaction | 1980-1983 | Poland | [112] |
| *Mycobacterium bovis*  (Tuberculosis) | *Cervus elaphus* | 9 | 72 | X |  | Culture | Reservoir | 2001-2002 | France | [13] |
|  |  | 33 | 138 | X |  | Culture | Reservoir | 2005-2006 | France | [13] |
|  |  | 86 | 543 | X |  | Gross lesions | Spill over | 1999-2004 | Spain | [122] |
|  |  | 1 |  | (case report) |  | Isolation | Unspecified | 1991 | Czech republic | [123] |
|  |  | 26 | 95 | X |  | Culture | Unspecified | 2006-2007 | Spain | [124] |
|  |  | 33 | 121* | X |  | Culture | Unspecified | 1996-2002 | Spain | [125] |
|  | *Capreolus capreolus* | 1 | 53 | X |  | Culture | Spillover |  | France | [13] |
|  | *Sus scrofa* | 25 | 85 | X |  | Culture | (£) | 2001-2002 | France | [13] |
|  |  | 65 | 155 | X |  | Culture | (£) | 2005-2006 | France | [13] |
|  |  | 269 | 474 | X |  | Gross lesions | Spill over | 1999-2004 | Spain | [122] |
|  |  | 51 | 96* | X |  | Culture | Unspecified | 1996-2002 | Spain | [125] |
|  |  | 65 | 126 | X |  | Culture | Reservoir | 2006-2007 | Spain | [124] |
|  |  | 3 |  | (case report) |  | Isolation | Unspecified | 1992 | Slovakia | [123] |
|  | *Dama dama* | 60 | 89* | X |  | Culture | Unspecified | 1996-2002 | Spain | [125] |
|  |  | 18 | 97 | X |  | Culture | Reservoir | 2006-2007 | Spain | [124] |
|  | *Bison bonasus* | 12 |  | (case report) |  | Isolation | Unspecified | 1997-1999 | Poland | [123] |
|  | *Capra aegragus** | 1 |  | (case report) |  | Isolation | Unspecified | 1991 | Czech republic | [123] |
|  | *Ammotragus lervia* | 33 | 67 |  | X | ELISA | Possible reservoir | 1999 | Spain | [126] |
| *Mycobacterium avium subsp paratuberculosis*  (Paratuberculosis) | *Alces alces* | 10 | 537 |  | X | ELISA | Unspecified | 1992-1999 | Norway | [127] |
|  | *Cervus elaphus* | 106 | 709* | X |  | RFLP | Unspecified | 1999-2001 | Czech Republic | [128] |
|  |  |  | 95 | 42.6 [95% CI : 32.6-52.6] |  | PCR | Unspecified | 2006-2007 | Spain | [129] |
|  |  | 257 | 852 |  | X | ELISA | Unspecified |  | Spain | [130] |
|  |  | 14 | 371 |  | X | ELISA | Unspecified | 1998 | Norway | [127] |
|  | *Ammotragus lervia* | 13 | 67 |  | X | ELISA | Possible reservoir | 1999 | Spain | [126] |
|  | *Dama dama* | 4 | 385* | X |  | RFLP | Unspecified | 1999-2001 | Czech Republic | [128] |
|  |  |  | 101 | 65.1 [95% CI : 55.1-75.1] |  | PCR | Unspecified | 2006-2007 | Spain | [131] |
|  |  | 1 | 94 | X |  | PCR | Unspecified | 2001-2003 | Spain | [131] |
|  |  | 2 | 5 | (case report) |  | PCR | Unspecified | 1997-1998 | Spain | [132] |
|  | *Ovis musimon* | 16 | 416* | X |  | RFLP | Unspecified | 1999-2001 | Czech Republic | [128] |
|  | *Capreolus capreolus* | 2 | 858 | X |  | RFLP | Unspecified | 1999-2001 | Czech Republic | [128] |
|  |  | 6 | 49 |  | X | ELISA | Unspecified | 1997 | Norway | [127] |
|  | *Rangifer tarandus* | 0 | 91 |  | X | ELISA | Unspecified | 1996 | Norway | [127] |
|  |  | 11 | 325 |  | X | ELISA | Unspecified | 1994 | Norway | [127] |
|  | *Sus scrofa* | 1 | 2 | X |  | RFLP | Accidental host | 1999-2001 | Czech Republic | [128] |
|  |  |  | 127 | 25.7 [95% CI : 17.6-33.8] |  | ELISA | Unspecified | 2006-2007 | Spain | [129] |
|  |  | 1 | 65 | X |  | PCR | Unspecified | 2001-2003 | Spain | [131] |
| *Mycoplasma conjunctivae* | *Capra ibex* | 16 | 136 | X |  | PCR | Possible carrier | 2006-2007 | Switzerland | [133] |
| *Mycoplasma agalactiae* | *Capra pyrenaica* | 46 | 422 | X |  | PCR | Unspecified | 1996-2003 | Spain | [134] |
| *Mycoplasma suis*  (Porcine infectious anemia) | *Sus scrofa* | 36 | 359 | X |  | PCR | Possible reservoir | 2007-2008 | Germany | [135] |
| *Mycoplasma hyopneumoniae* | *Sus scrofa* | 92 | 428 |  | X | ELISA | Not a reservoir | 2000-2008 | Spain | [136] |
| *Mannheimia sp*  (Pneumonia) | *Ovibos moschatus* | 71 | 276 | X |  | Isolation for some cases | Unspecified | 2006 | Norway | [137] |
| *Salmonella* spp*.* | *Ammotragus lervia* | 9 | 67 |  | X | Agglutination test | Unspecified | 1999 | Spain | [126] |
|  | *Sus scrofa* | 66 | 342 |  | X | ELISA | Unspecified | 2005-2006 | Italy | [110] |

Legend: n=number of positive animals; N=number of animals tested; CFT = Complement Fixation Test; IFA = Indirect immunofluorescence Assay; ELISA: Enzyme Linked Immuno Sorbent Assay; MAT = Microscopic Agglutination Test; PCR = Polymerase Chain Reaction; RBT = Rose Bengal Test; RFLP = restriction fragment length polymorphism method; * = animal from game park (isolated from the wild) or extensives farms; (&) = zoo animals; (£) = no epidemiological conclusion possible because of the sampling was not done randomly; (µ): 29 PCR were done on sera with positive serology; *(#):* foot necrobacillosis complex*= Fusobacterium necrophorum, Arcanobacter pyogenes, Streptococcus agalactiae, Staphylococcus aureus.*
