# Supplementary material for: A survey of the transmission of infectious diseases/infections between wild and domestic ungulates in Europe
Source: Vet Res. 2011 Jun 2;42(1):70. doi: 10.1186/1297-9716-42-70 (PMC3152899; doi:10.1186/1297-9716-42-70)
Supplement: Additional file 2 — [138-171]. Selected viral diseases reported in wild ungulates in Europe. This file is a table presenting a list of viral diseases already reported in wild ungulates in Europe. [file 1297-9716-42-70-S2.doc]

Additional file 2. Selected viral diseases reported in wild ungulates in Europe.

| **Pathogen** | **Ungulate specie**  **(latin name)** | **n** | **N** | **Prevalence** | **Sero-prevalence** | **Diagnostic method** | **Epidemiological role from author’s opinion** | **Year** | **Country** | **Reference** |
| --- | --- | --- | --- | --- | --- | --- | --- | --- | --- | --- |
| Aujezsky’s disease  virus (Pseudo-rabies) | *Sus scrofa* | 294 | 3143 |  | X | ELISA | Endemic | 1991-1994 | Germany | [138] |
|  |  | 9 | 16 |  | X | IFAT | Unspecified | 2000 | Spain | [139] |
|  |  | 101 | 338 |  | X | ELISA | Reservoir | 2004-2005 | Czech Republic | [140] |
|  |  | 92 | 929 |  | X | ELISA | Enzootic disease | 1993-2000 | Germany | [141] |
|  |  |  | 192 | 30,6 ± 6,7% |  | PCR | Widespread infection | 2004-2005 | Spain | [142] |
|  |  |  | 185 |  | 45.9 ± 7.8% | ELISA | Widespread infection | 2004-2005 | Spain | [142] |
|  |  | 111 | 427 |  | X | ELISA | Reservoir | 2003-2004 | Slovenia | [143] |
|  |  | 63 | 1857 |  | X | ELISA | Sporadic cases | 2001-2003 | Switzerland | [109] |
|  |  | 62 | 152 | X |  | PCR | Reservoir | 2002-2003 | Italy | [144] |
|  |  | 306 | 693 |  | X | ELISA | Unspecified | 2000-2003 | Spain | [145] |
|  |  | 0 | 24 |  | X | ELISA | Unspecified | 2004 | Lithuania | [146] |
|  |  | 423 | 12025 * |  | X | ELISA | Reservoir | 1991-1998 | France | [147] |
|  |  | 24 | 44 |  | X | ELISA | Reservoir | 1999 | Croatia | [148] |
|  |  | 105 | 342 |  | X | ELISA | Unspecified | 2005-2006 | Italy | [110] |
| African swine fever virus (African swine fever) | *Sus scrofa* | 14 | 147 |  | X | ELISA | Not a reservoir | 1991-1993 | Spain | [149] |
| Border disease virus  (Border disease) | *Sus scrofa* | 240 | 12025 * |  | X | ELISA | Unspecified | 1991-1998 | France | [147] |
|  | *Rupicapra pyrenaica* | 227 | 323 |  | X | ELISA | Unknown | 1994-2005 | France | [150] |
|  |  | 17 | 167 | X |  | ELISA+RT-PCR | Unknown | 1994-2005 | France | [150] |
|  |  | 82 | 114 |  | X | ELISA | Emerging disease | 2002-2006 | Spain | [151] |
|  |  | 10 | 10 (£) | X |  | RT-PCR | Emerging disease | 2005-2006 | Spain | [151] |
| Bovine herpes virus -1  (Infectious bovine rhinotracheitis) | *Rangifer tarandus* | 237 | 831 |  | X | VNT | Endemic disease | 1993-2000 | Norway | [152] |
|  | *Cervus elaphus* | 3 | 589 |  | X | VNT | Unspecified | 1993-2000 | Norway | [152] |
|  |  | 17 | 73 |  | X | VNT | Spill over | 2000-2002 | Germany | [153] |
|  | *Capreolus capreolus* | 18 | 602 |  | X | VNT | Unspecified | 1993-2000 | Norway | [152] |
|  |  | 4 | 38 |  | X | VNT | Spill over | 2000-2002 | Germany | [153] |
|  | *Dama dama* | 1 | 46 |  | X | VNT | Unspecified | 2000-2002 | Germany | [153] |
|  | *Bison bonasus* | 5 | 60 |  | X | ELISA | Unspecified | 1980-1983 | Poland | [112] |
|  | *Rupicapra pyrenaica ornata* | 7 | 27 |  | X | Microseroneutralisation | Unspecified | 1990-1993 | Italy | [154] |
| Bluetongue virus  (Blue Tongue) | *Cervus elaphus* |  | 513 |  | 40.4% | ID Screen Bluetongue  Competition assay | Unspecified | 2007 | Belgium | [37] |
|  |  | 309 | 1409 |  | X | ELISA | Unspecified | 2005-2007 | Spain | [38] |
|  | *Capreolus capreolus* | 2 | 39 |  | X | ELISA | Unspecified | 2005-2007 | Spain | [38] |
|  | *Dama dama* | 34 | 96 |  | X | ELISA | Unspecified | 2005-2007 | Spain | [38] |
|  | *Ovis aries* | 9 | 68 |  | X | ELISA | Unspecified | 2005-2007 | Spain | [38] |
|  |  | 4 | 6 | X (BTV-1) |  | RT-PCR | Unspecified | 2007 | Spain | [155] |
|  | *Ammotragus lervia* | 1 | 4 |  | X | ELISA | Unspecified | 2005-2007 | Spain | [38] |
|  | *Lama pacos** | 1 |  | (Case report) |  | PCR | Unspecified | 2007 | Germany | [156] |
| Bovine viral diarrhea virus  (Bovine viral diarrhea) | *Capra ibex* | 13 | 273 |  | X | ELISA | Unspecified | 2006-2008 | Switzerland | [115] |
|  | *Sus scrofa* | 2 | 352 |  | X | ELISA | Rarely exposed | 2004-2005 | Czech Republic | [140] |
|  |  | 2 | 44 |  | X | ELISA | Reservoir | 1999 | Croatia | [148] |
|  | *Rangifer tarandus* | 34 | 810 |  | X | VNT | Endemic disease | 1993-2000 | Norway | [152] |
|  | *Capreolus capreolus* | 78 | 635 |  | X | VNT | Endemic disease | 1993-2000 | Norway | [152] |
|  |  | 12 | 123 |  | X | VNT | Unspecified | 1990-1992 | Germany | [157] |
|  | *Cervus elaphus* | 7 | 658 |  | X | VNT | Unspecified | 1993-2000 | Norway | [152] |
|  |  | 2 | 20 |  | X | VNT | Unspecified | 1995-1996 | Denmark | [158] |
|  | *Alces alces* | 35 | 1794 |  | X | VNT | Unspecified | 1994-1999 | Norway | [152] |
| Caprine herpes virus -1  (Caprine herpesvirosis) | *Cervus elaphus* | 10 | 75 |  | X | VNT | Unspecified | 2000-2002 | Germany | [153] |
|  | *Capreolus capreolus* | 1 | 38 |  | X | VNT | Unspecified | 2000-2002 | Germany | [153] |
| Classic swine fever virus  (Classic swine fever) | *Sus scrofa* | 0 | 6471 |  | X | ELISA | Unspecified | 1999-2005 | Czech Republic | [140] |
|  |  | 28 | 1767 |  | X | ELISA | Sporadic cases | 2001-2003 | Switzerland | [109] |
|  |  | 0 | 591 |  | X | ELISA | Unspecified | 2004 | Lithuania | [144] |
|  |  | 585 | 5286 |  | X | ELISA | Reservoir | 2002-2004 | France | [159] |
|  |  | 128 | 301 |  | X | VNT | Reservoir | 2002-2004 | France | [159] |
|  |  | 96 | 2767 | X |  | PCR | Reservoir | 2002-2004 | France | [159] |
|  |  | 80 | 12025 * |  | X | ELISA | Unspecified | 1991-1998 | France | [147] |
|  |  | 17 | 44 |  | X | ELISA | Reservoir | 1999 | Croatia | [137] |
| Chronic wasting disease agent (Chronic wasting disease) | *Cervus elaphus* | 0 | 739 | X |  | ELISA | Unspecified | - | Italy | [160] |
|  |  | 0 | 674 | X |  | ELISA | Unspecified | 2001-2003 | Belgium | [161] |
|  | *Capreolus capreolus* | 0 | 192 | X |  | ELISA | Unspecified | 2001-2003 | Belgium | [161] |
| Encephalomyocarditis virus  (Encephalomycarditis) | *Sus scrofa* | 13 | 20 |  | X | VNT | Unspecified | 1994-2006 | Greece | [162] |
|  | *Rupicapra pyrenaica ornata* | 5 | 27 |  | X | Microseroneutralization | Unspecified | 1990-1993 | Italy | [154] |
| Foot and mouth disease virus  (Foot and mouth disease) | *Sus scrofa* | 0 | 504 |  | X | ELISA | Unspecified | 2004 | Lithuania | [146] |
|  |  | 0 | 208 |  | X | ELISA | Unspecified | 2001 | The Netherlands | [163] |
| Cervid herpes virus-1 | *Cervus elaphus* | 15 | 73 |  | X | VNT | Unspecified | 2000-2002 | Germany | [153] |
|  | *Capreolus capreolus* | 2 | 38 |  | X | VNT | Unspecified | 2000-2002 | Germany | [153] |
| Hepatite E virus  (Hepatitis E) | *Sus scrofa* | 143 | 676 |  | X | ELISA | Possible reservoir | 2001-2008 | Spain | [164] |
|  |  | 9 | 74 | X |  | RT-PCR | Possible reservoir | 2001-2006 | Hungary | [165] |
|  |  | 165 | 1039 |  | X | ELISA | Unspecified | 2005-2008 | The Netherlands | [166] |
|  |  | 8 | 106 | X |  | RT-PCR | Unspecified | 2005-2008 | The Netherlands | [166] |
|  | *Cervus elaphus* | 3 | 38 |  | X | ELISA | Unspecified | 2005-2008 | The Netherlands | [166] |
|  |  | 6 | 39 | X |  | RT-PCR | Unspecified | 2005-2008 | The Netherlands | [166] |
|  | *Capreolus capreolus* | 11 | 32 | X |  | RT-PCR | Possible reservoir | 2001-2006 | Hungary | [165] |
| Orf virus | *Ovibos moschatus* | 19 | 170 | X |  | Characterization | Spill-over | 2004 | Norway | [167] |
| Parapox virus  (Contagious echtyma) | *Rangifer tarandus** | 48 | 6 |  | X | Characterization | Unspecified | 2000 | Norway | [127] |
| Porcine circo virus -2  (Postweaning multisystemic wasting syndrome) | *Sus scrofa* | 57 | 134 |  | X | IFAT | Unspecified | 2005 | Czech Republic | [140] |
|  |  | 335 | 531 | X |  | Nested PCR | Unspecified | 2004-2007 | Germany | [168] |
|  |  | 314 | 656 |  | X | IPMA | Unspecified | 2000-2003 | Spain | [169] |
| Pestiviruses (unprecised)  (Pestivirus infections) | *Rupicapra pyrenaica ornata* | 6 | 35 * |  | X | ELISA | Unspecified | 1990-1993 | Italy | [154] |
|  | *Rupicapra rupicapra* | 28 | 110 |  | X | ELISA | Unspecified | 1999 | Italy | [170] |
|  |  | 145 | 343 |  | X | ELISA | Unspecified | 2004-2007 | France | [14] |
|  | *Cervus elaphus* | 8 | 136 |  | X | ELISA | Unspecified | 1999 | Italy | [170] |
|  | *Ovis amon* | 11 | 18 |  | X | ELISA | Unspecified | 2006-2007 | France | [14] |
|  | *Sus scrofa* | 7 | 56 |  | X | ELISA | Unspecified | 1999 | Italy | [170] |
| Porcine parvovirus (Porcine parvovirus infection) | *Sus scrofa* | 187 | 254 |  | X | HIT | Unspecified | 2004 | Lithuania | [146] |
|  |  | 27 | 342 |  | X | ELISA | Unspecified | 2005-2006 | Italy | [110] |
| Porcine reproductive and respiratory syndrome virus (porcine reproductive and respiratory syndrome) | *Sus scrofa* | 33 | 909 * |  | X | ELISA | Reservoir | 1991-1998 | France | [147] |
|  |  | 129 | 342 |  | X | ELISA | Unspecified | 2005-2006 | Italy | [110] |
| Small ruminant lentivirus | *Capra ibex* | 3 |  | (case report) |  | PCR | Independant cases | 2006 | France | [171] |
| Swine vesicular disease virus (Swine vesicular disease) | *Sus scrofa* | 0 | 12 |  | X | ELISA | Unspecified | 2004 | Lithuania | [146] |
| Transmissible gastroenteritis virus(Transmissible Gastroenteritis) | *Sus scrofa* | 1 | 134 |  | X | IFAT | Sporadic case | 2004-2005 | Czech Republic | [140] |

Legend: n: number of positive animals; N: number of animals tested; *: semi-domesticated animal or farmed animals; £: Only 10 animal tested, because were found sick or already dead in the field. Suspicion of the disease was present before doing the test; IFAT: Indirect Fluorescence Antibodies Test; ELISA: Enzyme Linked Immuno Sorbent Assay; HIT: Haemagglutination Inhibition Test; PCR: Polymerase Chain Reaction; RT-PCR: Reverse Transcriptase Polymerase Chain Reaction; VNT: Virus Neutralization Test.
