# Supplementary material for: A survey of the transmission of infectious diseases/infections between wild and domestic ungulates in Europe
Source: Vet Res. 2011 Jun 2;42(1):70. doi: 10.1186/1297-9716-42-70 (PMC3152899; doi:10.1186/1297-9716-42-70)
Supplement: Additional file 3 — [172-202]. Selected parasitic diseases reported in wild ungulates in Europe. This file is a table presenting a list of parasitic diseases already reported in wild ungulates in Europe. [file 1297-9716-42-70-S3.doc]

Additional file 3. Selected parasitic diseases reported in wild ungulates in Europe

| **Pathogen** | ***Ungulate specie***  ***(latin name)*** | **n** | **N** | **Prevalence** | **Serology** | **Diagnostic method** | **Epidemiological role from author’s opinion** | **Year** | **Country** | **Reference** |
| --- | --- | --- | --- | --- | --- | --- | --- | --- | --- | --- |
| **PROTOZOAN** |  |  |  |  |  |  |  |  |  |  |
| *Babesia capreoli* | *Rupicapra rupicapra* | 6 | 7 | (case report) |  | PCR | Emerging disease | 2005 | Switzerland | [41] |
|  |  | 1 | 48 | X |  | PCR | Unspecified | 2006-2007 | Switzerland | [172] |
|  | *Cervus elaphus* | 1 | 9 | X |  | PCR | Unspecified | 2006-2007 | Switzerland | [172] |
|  | *Capreolus capreolus* | 12 | 46 | X |  | PCR | Reservoir | 2006-2007 | Switzerland | [172] |
| *Babesia divergens* | *Rupicapra pyrenaica* |  |  | 15.79 [4.2-27.38] |  |  | Reservoir |  | Spain | [173] |
|  | *Capreolus capreolus* | 40 | 75 |  | X | Indirect IF | Unspecified | 1979 | France | [111] |
|  |  |  | 51 | 54.9 % |  | PCR | Zoonotic reservoir | 1996-2000 | Slovenia | [174] |
|  | *Cervus elaphus* |  | 30 | 16.7% |  | PCR | Zoonotic reservoir | 1996-2000 | Slovenia | [174] |
| *Babesia ovis* | *Ovis musimon* | 6 | 50 |  | X | IFAT | Reservoir | 1991-1996 | Spain | [175] |
|  | *Capra pyrenaica* | 155 | 475 |  | X | IFAT | Unspecified | 1992-1995 | Spain | [176] |
| *Babesia spp* | *Capra pyrenaica* | 1 | 1 | (case report) |  | Microscopic examination | Unspecified | 1995 | Spain | [177] |
|  | *Capreolus capreolus* |  |  | 53.3 [42.04-64.62] |  |  | Unspecified |  | France | [111] |
|  |  | 83 | 202 | X |  | PCR | Unspecified | 2004-2008 | France | [164] |
| *Babesia EU1* | *Capreolus capreolus* |  | 51 | 21.6% |  | PCR | Zoonotic reservoir | 1996-2000 | Slovenia | [174] |
| *Cryptosporidium spp.* | *Cervus elaphus* |  | 118* | 14,4 |  | IFA+PCR | Unspecified | 2003-2005 | Poland | [178] |
|  |  | 1 | 289 | X |  | Fecal examination | Reservoir | 2001-2003 | Norway | [179] |
|  | *Capreolus capreolus* |  | 22 | 9.1 |  | IFA+PCR | Unspecified | 2003 | Poland | [178] |
|  |  | 18 | 291 | X |  | Fecal examination | Reservoir | 2001-2003 | Norway | [179] |
|  | *Bison bonasus* |  | 55 | 29.1 |  | IFA+PCR | Unspecified | 2003-2005 | Poland | [178] |
|  | *Alces alces* | 15 | 455 | X |  | Fecal examination | Reservoir | 2001-2003 | Norway | [179] |
|  | *Sus scrofa* |  | 5 | 0 |  | IFA+PCR | Unspecified | 2003 | Poland | [178] |
| *Cryptosporidium parvum* | *Dama dama* | 1 | 16 | X |  | Fecal examination | Possible reservoir | 1995-1998 | England | [180] |
|  | *Muntiacus reevsi* | 4 | 42 | X |  | Fecal examination | Possible reservoir | 1995-1998 | England | [180] |
| *Dicrocoelium dendriticum* | *Capreolus capreolus* | 1 | 16 | X |  | H.E. | Unspecified | 1981-1998 | Belorussian Polesie | [181] |
|  | *Cervus elaphus* | 4 | 16 | X |  | H.E. | Unspecified | 1981-1998 | Belorussian Polesie | [181] |
| *Giardia spp.* | *Cervus elaphus* |  | 118* | 1,7 |  | IFA | Unspecified | 2003-2005 | Poland | [178] |
|  |  |  | 285 | 1 |  | Fecal examination | Unspecified |  | Croatia | [182] |
|  |  | 5 | 289 | X |  | Fecal examination | Reservoir | 2001-2003 | Norway | [179] |
|  | *Capreolus capreolus* |  | 22 | 4.5 |  | IFA | Unspecified | 2003 | Poland | [178] |
|  |  |  | 14 | 27 |  |  | Unspecified |  | Croatia | [183] |
|  |  | 45 | 291 | X |  | Fecal examination | Reservoir | 2001-2003 | Norway | [179] |
|  | *Bison bonasus* |  | 55 | 7.5 |  | IFA | Unspecified | 2003-2005 | Poland | [178] |
|  | *Alces alces* | 56 | 455 | X |  | Fecal examination | Reservoir | 2001-2003 | Norway | [179] |
|  |  | 1 | 1 | X |  | Fecal examination | Unspecified | 2002-2008 | Sweden | [184] |
|  | *Rangifer tarendus* | 11 | 155 | X |  | Fecal examination | Reservoir | 2001-2003 | Norway | [179] |
|  | *Sus scrofa* |  | 144 | 1.7 |  | Fecal examination | Unspecified |  | Croatia | [182] |
| *Neospora caninum* | *Ammotragus lervia* | 1 | 13 | X |  | ELISA + IFAT | Unspecified | 1993-2005 | Spain | [185] |
|  | *Capreolus capreolus* | 2 | 33 | X |  | ELISA + IFAT | Unspecified | 1993-2005 | Spain | [185] |
|  | *Cervus elaphus* | 28 | 237 | X |  | ELISA + IFAT | Unspecified | 1993-2005 | Spain | [185] |
|  | *Sus scrofa* | 1 | 298 | X |  | ELISA + IFAT | Unspecified | 1993-2005 | Spain | [185] |
|  |  | 102 | 565 | X |  | ELISA | Unspecified | 1999-2005 | Czech Republic | [186] |
| *Toxoplasma gondii* | *Alces alces* | 270 | 2142 | X |  | cDAT | Unspecified | 1992, 1994-2000 | Norway | [187] |
|  | *Cervus elaphus* | 1 | 67 |  | X | ELISA | Possible reservoir | 1999 | Spain | [126] |
|  |  | 1 | 10 | X |  | MAT | Unspecified | 1993-2005 | Spain | [188] |
|  | *Capra pyrenaica* | 1 | 3 | X |  | MAT | Unspecified | 1993-2005 | Spain | [188] |
|  | *Dama dama*  *Ovis ammon* | 12 | 32 | X |  | Isolation | Possible reservoir | 2003-2008 | France | [116] |
|  |  | 258 | 760 | X |  | cDAT | Unspecified | 1994, 1999-2000 | Norway | [187] |
|  |  | 7 | 33 | X |  | MAT | Unspecified | 1993-2005 | Spain | [188] |
|  | *Cervus elaphus* | 1 | 4 | X |  | Isolation | Possible reservoir | 2003-2008 | France | [116] |
|  |  | 69 | 441 | X |  | MAT | Possible source of zoonosis | 1993-2005 | Spain | [188] |
|  |  | 44 | 571 | X |  | cDAT | Unspecified | 1993-1999 | Norway | [187] |
|  | *Dama dama* | 18 | 79 | X |  | MAT | Unspecified | 1993-2005 | Spain | [188] |
|  | *Ovis ammon* | 4 | 27 | X |  | MAT | Unspecified | 1993-2005 | Spain | [188] |
|  | *Ovis gmelini* | 1 | 7 | X |  | Isolation | Possible reservoir | 2003-2008 | France | [116] |
|  | *Ovis orientalis musimon* | 17 | 77 |  | X | ELISA | Unspecified | - | Italy | [189] |
|  | *Rangifer tarandus* | 9 | 866 | X |  | cDAT | Unspecified | 1999-2000 | Norway | [187] |
|  | *Rupicapra rupicapra* | 2 | 10 | X |  | MAT | Unspecified | 1993-2005 | Spain | [188] |
|  | *Sus scrofa* | 26 | 148 | X |  | MAT | Reservoir | 2002-2008 | France | [190] |
|  |  | 148 | 565 | X |  | IFAT | Unspecified | 1999-2005 | Czech Republic | [186] |
| *Theileria sp. OT3* | *Cervus elaphus* |  |  | 85.7 |  |  | Reservoir |  | Spain | [173] |
|  | *Capreolus capreolus* |  |  | 46.4 |  |  | Reservoir |  | Spain | [173] |
|  | *Rupicapra pyrenaica* |  |  | 26.3 |  |  | Reservoir |  | Spain | [173] |
| *Theileria sp. 3185/02* | *Cervus elaphus* |  |  | 53.6 |  |  | Reservoir |  | Spain | [173] |
|  | *Capreolus capreolus* |  |  | 10.1 |  |  | Reservoir |  | Spain | [173] |
| *Sarcoptes scabei* | *Rupicapra rupicapra* | 1696 | 10000 | X |  |  | Unspecified | 1995-2004 | Italy | [191] |
|  | *Rupicapra pyrenaica parva* |  | 1600 | 12,9% |  | Observation | Unspecified | 1994-1995 | Spain | [192] |
|  | *Cervus elaphus* | 1 |  | (case report) |  | M.E. | Unspecified | 1995-2004 | Italy | [191] |
|  | *Capreolus capreolus* | 1 |  | (case report) |  | M.E. | Unspecified | 1995-2004 | Italy | [191] |
|  | *Ovis gmelini musimon* | 1 |  | (case report) |  | M.E. | Unspecified | 1995-2004 | Italy | [191] |
|  | *Capra pyrenaica* |  | 2096 | 49.2 ± 7.9 |  | M.E. | Unspecified | 1995-2006 | Spain | [193] |
|  | *Rupicapra pyrenaica* | 43 | 63 | X |  | M.E. | Unspecified | 1988 | Spain | [194] |
|  | *Capra ibex* | 157 |  | (case report) |  | M.E. | Unspecified | 1995-2006 | Italy | [195] |
|  | *Spanish ibex* |  |  | 100 (epizoology) | - | histopathology | New infection of a naive population |  | Spain | [194] |
| *TREMATODA* |  |  |  |  |  |  |  |  |  |  |
| *Dicrocoelium dendriticum* | *Capreolus capreolus* | 1 | 16 | X |  | H.E. | Unspecified | 1981-1998 | Belorussian Polesie | [181] |
|  | *Cervus elaphus* | 4 | 16 | X |  | H.E. | Unspecified | 1981-1998 | Belorussian Polesie | [181] |
| *Fasciola hepatica* | *Capra pyrenaica* | 10 | 2096$ | X |  | Necropsy | Unspecified | 1995-2006 | Spain | [193] |
|  |  | 5 | 380$ | X |  | Coprology | Unspecified | 1995-2006 | Spain | [193] |
|  | *Cervus elaphus* | 5 | 16 | X |  | H.E. | Unspecified | 1981-1998 | Belorussian Polesie | [181] |
|  | *Capreolus capreolus* | 1 | 1 | Case report |  | Necropsy | Unspecified | 2006 | France | [196] |
|  |  | 1 | 16 | X |  | H.E. | Unspecified | 1981-1998 | Belorussian Polesie | [181] |
|  | *Alces alces* | 1 | 18 | X |  | H.E. | Unspecified | 1981-1998 | Belorussian Polesie | [181] |
| *Fascioloides magna* | *Cervus elaphus* |  |  | Case report |  | Necropsy | Unspecified |  | Croatia | [197] |
| *Liorchis scotiae* | *Alces alces* | 4 | 18 | X |  | H.E. | Unspecified | 1981-1998 | Belorussian Polesie | [181] |
| *Parafasciolopsis fasciolaemorpha* | *Alces alces* | 8 | 18 | X |  | H.E. | Unspecified | 1981-1998 | Belorussian Polesie | [181] |
|  | *Capreolus capreolus* | 2 | 16 | X |  | H.E. | Unspecified | 1981-1998 | Belorussian Polesie | [181] |
| *Paramphistomum cervi* | *Cervus elaphus* | 3 | 16 | X |  | H.E. | Unspecified | 1981-1998 | Belorussian Polesie | [181] |
| *Paramphistomum ichikawai* | *Alces alces* | 6 | 18 | X |  | H.E. | Unspecified | 1981-1998 | Belorussian Polesie | [181] |
|  | *Capreolus capreolus* | 2 | 16 | X |  | H.E. | Unspecified | 1981-1998 | Belorussian Polesie | [181] |
| *CESTODA* |  |  |  |  |  |  |  |  |  |  |
| *Echinococcus granulosus* | *Alces alces* | 3 | 18 | X |  | H.E. | Unspecified | 1981-1998 | Belorussian Polesie | [181] |
|  | *Cervus elaphus* | 3 | 16 | X |  | H.E. | Unspecified | 1981-1998 | Belorussian Polesie | [181] |
| *Moniezia benedeni* | *Alces alces* | 5 | 18 | X |  | H.E. | Unspecified | 1981-1998 | Belorussian Polesie | [181] |
| *Taenia hydatigena* | *Alces alces* | 8 | 18 | X |  | H.E. | Unspecified | 1981-1998 | Belorussian Polesie | [181] |
|  | *Capreolus capreolus* | 1 | 16 | X |  | H.E. | Unspecified | 1981-1998 | Belorussian Polesie | [181] |
|  | *Cervus elaphus* | 2 | 16 | X |  | H.E. | Unspecified | 1981-1998 | Belorussian Polesie | [181] |
| *Taenia krabbei* | *Cervus elaphus* | 2 | 16 | X |  | H.E. | Unspecified | 1981-1998 | Belorussian Polesie | [181] |
| ***NEMATODA*** |  |  |  |  |  |  |  |  |  |  |
| *Bunostomum trigonocephalum* | *Alces alces* | 5 | 18 | X |  | H.E. | Unspecified | 1981-1998 | Belorussian Polesie | [181] |
| *Chabertia ovina* | *Capreolus capreolus* | 8 | 16 | X |  | H.E. | Unspecified | 1981-1998 | Belorussian Polesie | [181] |
| *Dictyocaulus eckerti* | *Alces alces* | 4 | 18 | X |  | H.E. | Unspecified | 1981-1998 | Belorussian Polesie | [181] |
|  | *Capreolus capreolus* | 2 | 16 | X |  | H.E. | Unspecified | 1981-1998 | Belorussian Polesie | [181] |
|  | *Cervus elaphus* | 9 | 16 | X |  | H.E. | Unspecified | 1981-1998 | Belorussian Polesie | [181] |
| *Nematodirus oiratianus* | *Cervus elaphus* | 4 | 16 | X |  | H.E. | Unspecified | 1981-1998 | Belorussian Polesie | [181] |
| *Oesophagostomum venulosum* | *Alces alces* | 3 | 18 | X |  | H.E. | Unspecified | 1981-1998 | Belorussian Polesie | [181] |
|  | *Capreolus capreolus* | 5 | 18 | X |  | H.E. | Unspecified | 1981-1998 | Belorussian Polesie | [181] |
|  | *Cervus elaphus* | 5 | 16 | X |  | H.E. | Unspecified | 1981-1998 | Belorussian Polesie | [181] |
| *Onchocerca flexuosa* | *Cervus elaphus* | 10 | 16 | X |  | H.E. | Unspecified | 1981-1998 | Belorussian Polesie | [181] |
| *Setaria cervi* | *Capreolus capreolus* | 4 | 18 | X |  | H.E. | Unspecified | 1981-1998 | Belorussian Polesie | [181] |
| *Trichuris ovis* | *Alces alces* | 6 | 18 | X |  | H.E. | Unspecified | 1981-1998 | Belorussian Polesie | [181] |
|  | *Capreolus capreolus* | 6 | 18 | X |  | H.E. | Unspecified | 1981-1998 | Belorussian Polesie | [181] |
|  | *Cervus elaphus* | 5 | 16 | X |  | H.E. | Unspecified | 1981-1998 | Belorussian Polesie | [181] |
| *Trichinella spp.* | *Sus scrofa* | 13 | 1035 |  | X | ELISA | Unspecified | 2003-2004 | Slovak republic | [198] |
|  |  | 30 | 1492 | X |  | ELISA | Unspecified | 2006-2008 | France | [199] |
| *Trichinella britovi* | *Sus scrofa* | 1 | 1 |  | Case report | Artificial digestion + PCR | Unspecified | 2004 | Belgium | [200] |
|  |  | 3 | 3 |  | Cases report | Artificial digestion + PCR | Unspecified | Unspecified | Roumania | [201] |
| *Trichinella spiralis* | *Sus scrofa* | 2 | 2 |  | Case report | Artificial digestion + PCR | Unspecified | Unspecified | Roumania | [201] |
|  |  | - | 458 |  | 6.8% | ELISA | Unpecified | Unspecified | The Netherlands | [202] |
| *Toxocara spp.* | *Sus scrofa* | 85 | 1173 |  | X | ELISA | Unspecified | 2003-2004 | Slovak republic | [198] |
| *Ascaris suum* | *Sus scrofa* | 45 | 411 |  | X | ELISA | Unspecified | 2003-2004 | Slovak republic | [198] |

Legend: *: farmed animals; $: same study total of 2096 ibexes: all were analysed by necropsy and 380 of them were additionally analysed by coprology; cDAT: commercial Direct Agglutination Test; ELISA: Enzyme Linked Immuno Sorbent Assay; IF: immunofluorescence; IFAT: Indirect Fluorescent Antibody Test; H.E.: Helminthological Examination: dissection and organ compression; MAT: Modified Agglutination Test; M.E.: Microscopic Examination; PCR: Polymerase Chain Reaction.
